# Supplementary material for: Creating a Research-Ready Data Asset version of primary care data for Wales and investigating the impact of COVID-19 on utilisation of primary care services
Source: PLoS One. 2025 Dec 10;20(12):e0338652. doi: 10.1371/journal.pone.0338652 (PMC12694842; doi:10.1371/journal.pone.0338652)
Supplement: S1 File — (DOCX) [file pone.0338652.s001.docx]

**Illustrative examples of clinical code standardisation and duplicate resolution in the WLGP RRDA clinical code look-up**

The following examples illustrate how potential duplicate clinical codes across different sources were handled during the creation of the WLGP RRDA clinical code look-up. For each pair of codes, we show the original code, its code type, the standardised format applied, any format adjustment performed (such as truncation or padding), and the final status indicating whether the code was retained or removed in the final look-up. These examples demonstrate the application of the predefined prioritisation hierarchy across code types—official Read V2 codes, official SNOMED codes, DHCW local Vision codes, DHCW local EMIS codes, additional Read or Vision codes, and additional EMIS codes from the UK Biobank—as well as the standardisation procedures used in Step 5 of the WLGP RRDA development section.

*Example 1: Resolving duplicates between a Read V2 code and an additional Read/Vision code.*

| **Original code** | **Code type** | **Standardised Code** | **Format adjustment** | **Status** |
| --- | --- | --- | --- | --- |
| 1234. | Official Read V2 | 1234. | Valid format | Kept |
| 1234 | Additional Read or Vision codes | 1234. | Padded with “.” | Removed |

*Example 2: Resolving duplicates between a SNOMED code and a DHCW Vision code.*

| **Original code** | **Code type** | **Standardised Code** | **Format adjustment** | **Status** |
| --- | --- | --- | --- | --- |
| 5678900 | SNOMED | 5678900 | Valid format | Kept |
| 5678900 | DHCW Vision | 56789 | Truncated to 5 characters | Kept |

*Example 3: Resolving duplicates between a DHCW EMIS code and an additional Read/Vision code.*

| **Original code** | **Code type** | **Standardised Code** | **Format adjustment** | **Status** |
| --- | --- | --- | --- | --- |
| ABCD | DHCW EMIS | ABCD | Valid format | Kept |
| ABCD | Additional Read or Vision codes | ABCD. | Padded with “.” | Kept |

*Example 4: Resolving duplicates between a Read V2 code and a DHCW EMIS code.*

| **Original code** | **Code type** | **Standardised Code** | **Format adjustment** | **Status** |
| --- | --- | --- | --- | --- |
| ABC12 | Official Read V2 | ABC12 | Valid format | Kept |
| ABC12 | DHCW EMIS | ABC12 | Valid format | Removed |

*Example 5: Resolving duplicates between a DHCW EMIS code and a UK Biobank EMIS code.*

| **Original code** | **Code type** | **Standardised Code** | **Format adjustment** | **Status** |
| --- | --- | --- | --- | --- |
| EMIS1234 | DHCW EMIS | EMIS1234 | Valid format | Kept |
| EMIS1234 | UK Biobank EMIS | EMIS1234 | Valid format | Removed |

*Example 6: Resolving duplicates between a DHCW Vision code and a DHCW EMIS code.*

| **Original code** | **Code type** | **Standardised Code** | **Format adjustment** | **Status** |
| --- | --- | --- | --- | --- |
| 1234511 | DHCW Vision | 12345 | Truncated to 5 characters | Kept |
| 12345 | DHCW EMIS | 12345 | Valid format | Removed |
