# Supplementary material for: Creating a Research-Ready Data Asset version of primary care data for Wales and investigating the impact of COVID-19 on utilisation of primary care services
Source: PLoS One. 2025 Dec 10;20(12):e0338652. doi: 10.1371/journal.pone.0338652 (PMC12694842; doi:10.1371/journal.pone.0338652)
Supplement: S1 Table — (DOCX) [file pone.0338652.s002.docx]

| **Year** | 1990 | 1995 | 2000 | 2005 | 2010 | 2015 | 2020 | 2024 |
| --- | --- | --- | --- | --- | --- | --- | --- | --- |
| **Total** | 2,137,980 (100%) | 2,903,630 (100%) | 2,962,900 (100%) | 3,049,370 (100%) | 3,106,980 (100%) | 3,130,210 (100%) | 3,199,040 (100%) | 3,237,480 (100%) |
| **Sex** |  |  |  |  |  |  |  |  |
| Male | 1,056,340 (49.4%) | 1,426,450 (49.1%) | 1,465,740 (49.5%) | 1,514,740 (49.7%) | 1,552,580 (50.0%) | 1,565,820 (50.0%) | 1,598,170 (50.0%) | 1,616,100 (49.9%) |
| Female | 1,081,650 (50.6%) | 1,477,180 (50.9%) | 1,497,160 (50.5%) | 1,534,640 (50.3%) | 1,554,400 (50.0%) | 1,564,390 (50.0%) | 1,600,880 (50.0%) | 1,621,380 (50.1%) |
| **Age** |  |  |  |  |  |  |  |  |
| 0-15 | 450,390 (21.1%) | 589,900 (20.3%) | 580,210 (19.6%) | 555,070 (18.2%) | 540,510 (17.4%) | 539,070 (17.2%) | 549,640 (17.2%) | 536,470 (16.6%) |
| 16-34 | 491,490 (23.0%) | 747,460 (25.7%) | 736,080 (24.8%) | 744,850 (24.4%) | 754,510 (24.3%) | 750,740 (24.0%) | 737,040 (23.0%) | 731,260 (22.6%) |
| 35-49 | 453,750 (21.2%) | 588,720 (20.3%) | 607,870 (20.5%) | 649,980 (21.3%) | 652,290 (21.0%) | 605,470 (19.3%) | 592,660 (18.5%) | 608,840 (18.8%) |
| 50-64 | 385,480 (18.0%) | 477,680 (16.5%) | 534,630 (18.0%) | 577,520 (18.9%) | 600,340 (19.3%) | 613,000 (19.6%) | 653,920 (20.4%) | 663,370 (20.5%) |
| 65-110 | 356,880 (16.7%) | 499,880 (17.2%) | 504,110 (17.0%) | 521,950 (17.1%) | 559,330 (18.0%) | 621,930 (19.9%) | 665,790 (20.8%) | 697,520 (21.5%) |
| **WIMD 2019 Quintile** |  |  |  |  |  |  |  |  |
| 1 (Most) | 455,760 (21.3%) | 621,680 (21.4%) | 603,620 (20.4%) | 606,370 (19.9%) | 612,760 (19.7%) | 623,260 (19.9%) | 644,840 (20.2%) | 654,490 (20.2%) |
| 2 | 442,430 (20.7%) | 589,710 (20.3%) | 592,870 (20.0%) | 602,200 (19.7%) | 614,220 (19.8%) | 617,100 (19.7%) | 629,930 (19.7%) | 635,650 (19.6%) |
| 3 | 432,090 (20.2%) | 589,530 (20.3%) | 603,620 (20.4%) | 621,550 (20.4%) | 633,000 (20.4%) | 635,610 (20.3%) | 649,690 (20.3%) | 658,710 (20.3%) |
| 4 | 422,990 (19.8%) | 570,040 (19.6%) | 588,200 (19.9%) | 612,470 (20.1%) | 625,590 (20.1%) | 629,440 (20.1%) | 642,140 (20.1%) | 649,430 (20.1%) |
| 5 (Least) | 384,710 (18.0%) | 532,660 (18.3%) | 574,590 (19.4%) | 606,790 (19.9%) | 621,410 (20.0%) | 624,820 (20.0%) | 632,460 (19.8%) | 639,180 (19.7%) |
| **Health Board** |  |  |  |  |  |  |  |  |
| Aneurin Bevan | 413,390 (19.3%) | 552,060 (19.0%) | 559,990 (18.9%) | 576,860 (18.9%) | 584,540 (18.8%) | 587,880 (18.8%) | 606,590 (19.0%) | 616,000 (19.0%) |
| Betsi Cadwaladr | 489,100 (22.9%) | 653,030 (22.5%) | 664,980 (22.4%) | 687,350 (22.5%) | 696,840 (22.4%) | 696,550 (22.3%) | 704,090 (22.0%) | 705,900 (21.8%) |
| Cardiff and Vale | 308,580 (14.4%) | 433,040 (14.9%) | 454,830 (15.4%) | 467,340 (15.3%) | 487,130 (15.7%) | 502,210 (16.0%) | 520,430 (16.3%) | 532,890 (16.5%) |
| Cwm Taf Morgannwg | 308,430 (14.4%) | 424,780 (14.6%) | 426,720 (14.4%) | 432,810 (14.2%) | 439,710 (14.2%) | 441,440 (14.1%) | 453,110 (14.2%) | 455,560 (14.1%) |
| Hywel Dda | 261,000 (12.2%) | 351,140 (12.1%) | 358,750 (12.1%) | 373,930 (12.3%) | 381,600 (12.3%) | 381,840 (12.2%) | 389,060 (12.2%) | 394,600 (12.2%) |
| Powys | 83,390 (3.9%) | 118,130 (4.1%) | 123,970 (4.2%) | 130,370 (4.3%) | 133,000 (4.3%) | 132,770 (4.2%) | 133,130 (4.2%) | 135,070 (4.2%) |
| Swansea Bay | 274,110 (12.8%) | 371,440 (12.8%) | 373,650 (12.6%) | 380,720 (12.5%) | 384,150 (12.4%) | 387,550 (12.4%) | 392,660 (12.3%) | 397,460 (12.3%) |
