# Supplementary material for: Creating a Research-Ready Data Asset version of primary care data for Wales and investigating the impact of COVID-19 on utilisation of primary care services
Source: PLoS One. 2025 Dec 10;20(12):e0338652. doi: 10.1371/journal.pone.0338652 (PMC12694842; doi:10.1371/journal.pone.0338652)
Supplement: S2 Table — (DOCX) [file pone.0338652.s003.docx]

| **Year** | 1990 | 1995 | 2000 | 2005 | 2010 | 2015 | 2020 | 2024 |
| --- | --- | --- | --- | --- | --- | --- | --- | --- |
| **Total** | 752,170 (100%) | 2,285,920 (100%) | 2,522,520 (100%) | 2,687,000 (100%) | 2,750,500 (100%) | 2,762,530 (100%) | 2,796,090 (100%) | 2,792,110 (100%) |
| **Sex** |  |  |  |  |  |  |  |  |
| Male | 351,910 (46.8%) | 1,103,730 (48.3%) | 1,240,450 (49.2%) | 1,331,060 (49.5%) | 1,371,640 (49.9%) | 1,378,890 (49.9%) | 1,393,740 (49.8%) | 1,390,220 (49.8%) |
| Female | 400,260 (53.2%) | 1,182,190 (51.7%) | 1,282,070 (50.8%) | 1,355,950 (50.5%) | 1,378,860 (50.1%) | 1,383,640 (50.1%) | 1,402,360 (50.2%) | 1,401,890 (50.2%) |
| **Age** |  |  |  |  |  |  |  |  |
| 0-15 | 169,900 (22.6%) | 492,640 (21.6%) | 511,670 (20.3%) | 498,060 (18.5%) | 484,580 (17.6%) | 479,490 (17.4%) | 484,510 (17.3%) | 467,170 (16.7%) |
| 16-34 | 182,730 (24.3%) | 596,700 (26.1%) | 633,900 (25.1%) | 671,920 (25.0%) | 680,630 (24.7%) | 671,930 (24.3%) | 654,350 (23.4%) | 638,920 (22.9%) |
| 35-49 | 170,450 (22.7%) | 478,230 (20.9%) | 523,800 (20.8%) | 574,150 (21.4%) | 578,640 (21.0%) | 535,930 (19.4%) | 519,640 (18.6%) | 526,640 (18.9%) |
| 50-64 | 133,580 (17.8%) | 377,490 (16.5%) | 452,030 (17.9%) | 501,850 (18.7%) | 524,940 (19.1%) | 535,530 (19.4%) | 565,380 (20.2%) | 567,580 (20.3%) |
| 65-110 | 95,520 (12.7%) | 340,870 (14.9%) | 401,110 (15.9%) | 441,020 (16.4%) | 481,700 (17.5%) | 539,640 (19.5%) | 572,220 (20.5%) | 591,780 (21.2%) |
| **WIMD 2019 Quintile** |  |  |  |  |  |  |  |  |
| 1 (Most) | 166,980 (22.2%) | 496,180 (21.7%) | 523,740 (20.8%) | 547,970 (20.4%) | 558,330 (20.3%) | 569,180 (20.6%) | 583,140 (20.9%) | 583,750 (20.9%) |
| 2 | 163,980 (21.8%) | 474,920 (20.8%) | 515,670 (20.4%) | 545,220 (20.3%) | 558,950 (20.3%) | 556,510 (20.1%) | 562,140 (20.1%) | 558,000 (20.0%) |
| 3 | 146,420 (19.5%) | 456,860 (20.0%) | 506,320 (20.1%) | 539,680 (20.1%) | 553,400 (20.1%) | 554,200 (20.1%) | 561,460 (20.1%) | 561,730 (20.1%) |
| 4 | 141,800 (18.9%) | 428,990 (18.8%) | 476,750 (18.9%) | 510,090 (19.0%) | 522,740 (19.0%) | 524,670 (19.0%) | 530,140 (19.0%) | 529,340 (19.0%) |
| 5 (Least) | 132,990 (17.7%) | 428,970 (18.8%) | 500,040 (19.8%) | 544,050 (20.2%) | 557,080 (20.3%) | 557,980 (20.2%) | 559,220 (20.0%) | 559,280 (20.0%) |
| **Health Board** |  |  |  |  |  |  |  |  |
| Aneurin Bevan | 134,670 (17.9%) | 422,120 (18.5%) | 465,780 (18.5%) | 499,050 (18.6%) | 506,940 (18.4%) | 508,910 (18.4%) | 518,390 (18.5%) | 518,780 (18.6%) |
| Betsi Cadwaladr | 160,430 (21.3%) | 478,680 (20.9%) | 535,260 (21.2%) | 579,740 (21.6%) | 589,090 (21.4%) | 588,890 (21.3%) | 593,110 (21.2%) | 585,020 (21.0%) |
| Cardiff and Vale | 132,040 (17.6%) | 363,190 (15.9%) | 410,220 (16.3%) | 434,520 (16.2%) | 453,920 (16.5%) | 465,640 (16.9%) | 473,450 (16.9%) | 479,870 (17.2%) |
| Cwm Taf Morgannwg | 175,380 (23.3%) | 374,480 (16.4%) | 403,650 (16.0%) | 424,090 (15.8%) | 435,220 (15.8%) | 439,290 (15.9%) | 452,050 (16.2%) | 454,570 (16.3%) |
| Hywel Dda | 71,820 (9.5%) | 268,820 (11.8%) | 297,980 (11.8%) | 316,790 (11.8%) | 328,120 (11.9%) | 324,830 (11.8%) | 323,440 (11.6%) | 324,480 (11.6%) |
| Powys | 8,720 (1.2%) | 53,670 (2.3%) | 59,480 (2.4%) | 63,400 (2.4%) | 64,120 (2.3%) | 62,120 (2.2%) | 60,060 (2.1%) | 58,910 (2.1%) |
| Swansea Bay | 69,110 (9.2%) | 324,950 (14.2%) | 350,140 (13.9%) | 369,430 (13.7%) | 373,090 (13.6%) | 372,870 (13.5%) | 375,600 (13.4%) | 370,490 (13.3%) |
