# Supplementary material for: Creating a Research-Ready Data Asset version of primary care data for Wales and investigating the impact of COVID-19 on utilisation of primary care services
Source: PLoS One. 2025 Dec 10;20(12):e0338652. doi: 10.1371/journal.pone.0338652 (PMC12694842; doi:10.1371/journal.pone.0338652)
Supplement: S3 Table — (DOCX) [file pone.0338652.s004.docx]

| **Year** | 1990 | 1995 | 2000 | 2005 | 2010 | 2015 | 2020 | 2024 |
| --- | --- | --- | --- | --- | --- | --- | --- | --- |
| **Total** | 2,137,980 (35.2%) | 2,903,630 (78.7%) | 2,962,900 (85.1%) | 3,049,370 (88.1%) | 3,106,980 (88.5%) | 3,130,210 (88.3%) | 3,199,040 (87.4%) | 3,237,480 (86.2%) |
| **Sex** |  |  |  |  |  |  |  |  |
| Male | 1,056,340 (33.3%) | 1,426,450 (77.4%) | 1,465,740 (84.6%) | 1,514,740 (87.9%) | 1,552,580 (88.3%) | 1,565,820 (88.1%) | 1,598,170 (87.2%) | 1,616,100 (86.0%) |
| Female | 1,081,650 (37.0%) | 1,477,180 (80.0%) | 1,497,160 (85.6%) | 1,534,640 (88.4%) | 1,554,400 (88.7%) | 1,564,390 (88.4%) | 1,600,880 (87.6%) | 1,621,380 (86.5%) |
| **Age** |  |  |  |  |  |  |  |  |
| 0-15 | 450,390 (37.7%) | 589,900 (83.5%) | 580,210 (88.2%) | 555,070 (89.7%) | 540,510 (89.7%) | 539,070 (88.9%) | 549,640 (88.2%) | 536,470 (87.1%) |
| 16-34 | 491,490 (37.2%) | 747,460 (79.8%) | 736,080 (86.1%) | 744,850 (90.2%) | 754,510 (90.2%) | 750,740 (89.5%) | 737,040 (88.8%) | 731,260 (87.4%) |
| 35-49 | 453,750 (37.6%) | 588,720 (81.2%) | 607,870 (86.2%) | 649,980 (88.3%) | 652,290 (88.7%) | 605,470 (88.5%) | 592,660 (87.7%) | 608,840 (86.5%) |
| 50-64 | 385,480 (34.7%) | 477,680 (79.0%) | 534,630 (84.6%) | 577,520 (86.9%) | 600,340 (87.4%) | 613,000 (87.4%) | 653,920 (86.5%) | 663,370 (85.6%) |
| 65-110 | 356,880 (26.8%) | 499,880 (68.2%) | 504,110 (79.6%) | 521,950 (84.5%) | 559,330 (86.1%) | 621,930 (86.8%) | 665,790 (85.9%) | 697,520 (84.8%) |
| **WIMD 2019 Quintile** |  |  |  |  |  |  |  |  |
| 1 (Least deprived) | 455,760 (36.6%) | 621,680 (79.8%) | 603,620 (86.8%) | 606,370 (90.4%) | 612,760 (91.1%) | 623,260 (91.3%) | 644,840 (90.4%) | 654,490 (89.2%) |
| 2 | 442,430 (37.1%) | 589,710 (80.5%) | 592,870 (87.0%) | 602,200 (90.5%) | 614,220 (91.0%) | 617,100 (90.2%) | 629,930 (89.2%) | 635,650 (87.8%) |
| 3 | 432,090 (33.9%) | 589,530 (77.5%) | 603,620 (83.9%) | 621,550 (86.8%) | 633,000 (87.4%) | 635,610 (87.2%) | 649,690 (86.4%) | 658,710 (85.3%) |
| 4 | 422,990 (33.5%) | 570,040 (75.3%) | 588,200 (81.1%) | 612,470 (83.3%) | 625,590 (83.6%) | 629,440 (83.4%) | 642,140 (82.6%) | 649,430 (81.5%) |
| 5 (Most deprived) | 384,710 (34.6%) | 532,660 (80.5%) | 574,590 (87.0%) | 606,790 (89.7%) | 621,410 (89.6%) | 624,820 (89.3%) | 632,460 (88.4%) | 639,180 (87.5%) |
| **Health Board** |  |  |  |  |  |  |  |  |
| Aneurin Bevan | 413,390 (32.6%) | 552,060 (76.5%) | 559,990 (83.2%) | 576,860 (86.5%) | 584,540 (86.7%) | 587,880 (86.6%) | 606,590 (85.5%) | 616,000 (84.2%) |
| Betsi Cadwaladr | 489,100 (32.8%) | 653,030 (73.3%) | 664,980 (80.5%) | 687,350 (84.3%) | 696,840 (84.5%) | 696,550 (84.5%) | 704,090 (84.2%) | 705,900 (82.9%) |
| Cardiff and Vale | 308,580 (42.8%) | 433,040 (83.9%) | 454,830 (90.2%) | 467,340 (93.0%) | 487,130 (93.2%) | 502,210 (92.7%) | 520,430 (91.0%) | 532,890 (90.1%) |
| Cwm Taf Morgannwg | 308,430 (56.9%) | 424,780 (88.2%) | 426,720 (94.6%) | 432,810 (98.0%) | 439,710 (99.0%) | 441,440 (99.5%) | 453,110 (99.8%) | 455,560 (99.8%) |
| Hywel Dda | 261,000 (27.5%) | 351,140 (76.6%) | 358,750 (83.1%) | 373,930 (84.7%) | 381,600 (86.0%) | 381,840 (85.1%) | 389,060 (83.1%) | 394,600 (82.2%) |
| Powys | 83,390 (10.5%) | 118,130 (45.4%) | 123,970 (48.0%) | 130,370 (48.6%) | 133,000 (48.2%) | 132,770 (46.8%) | 133,130 (45.1%) | 135,070 (43.6%) |
| Swansea Bay | 274,110 (25.2%) | 371,440 (87.5%) | 373,650 (93.7%) | 380,720 (97.0%) | 384,150 (97.1%) | 387,550 (96.2%) | 392,660 (95.7%) | 397,460 (93.2%) |
