# Supplementary figures and images for: Creating a Research-Ready Data Asset version of primary care data for Wales and investigating the impact of COVID-19 on utilisation of primary care services
Source: PLoS One. 2025 Dec 10;20(12):e0338652. doi: 10.1371/journal.pone.0338652 (PMC12694842; doi:10.1371/journal.pone.0338652)

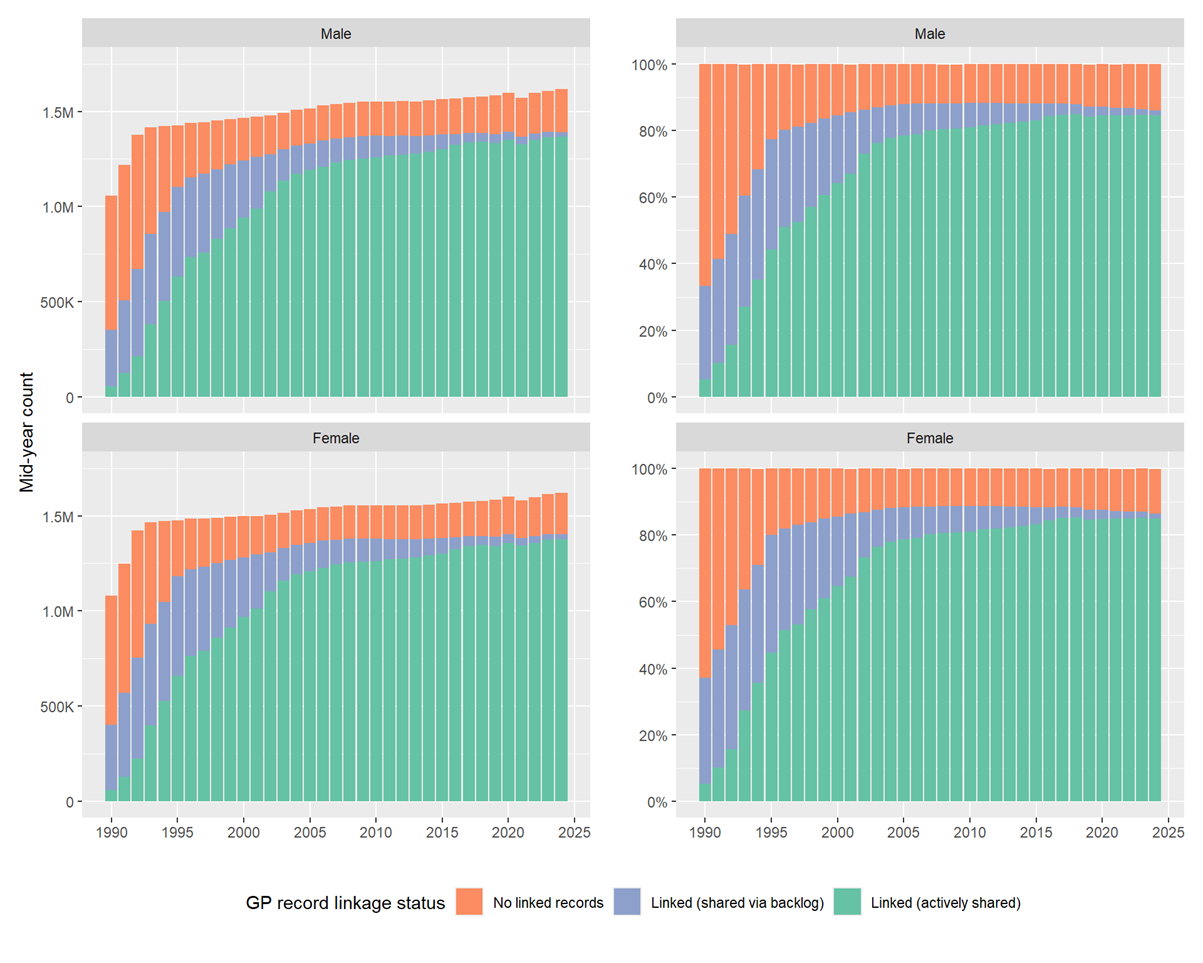

Supplement: S1 Fig — (TIF) [file pone.0338652.s005.tif]

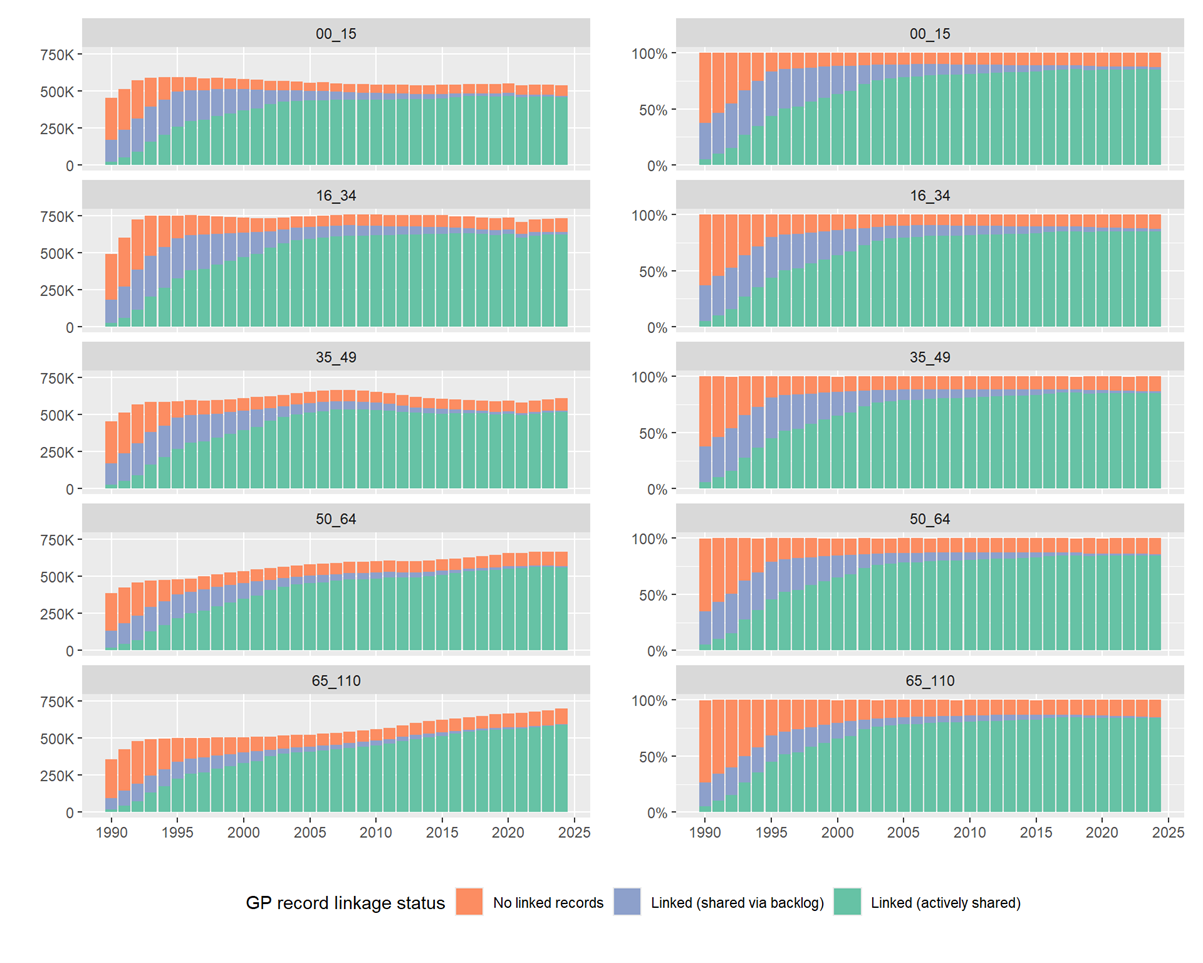

Supplement: S2 Fig — (TIF) [file pone.0338652.s006.tif]

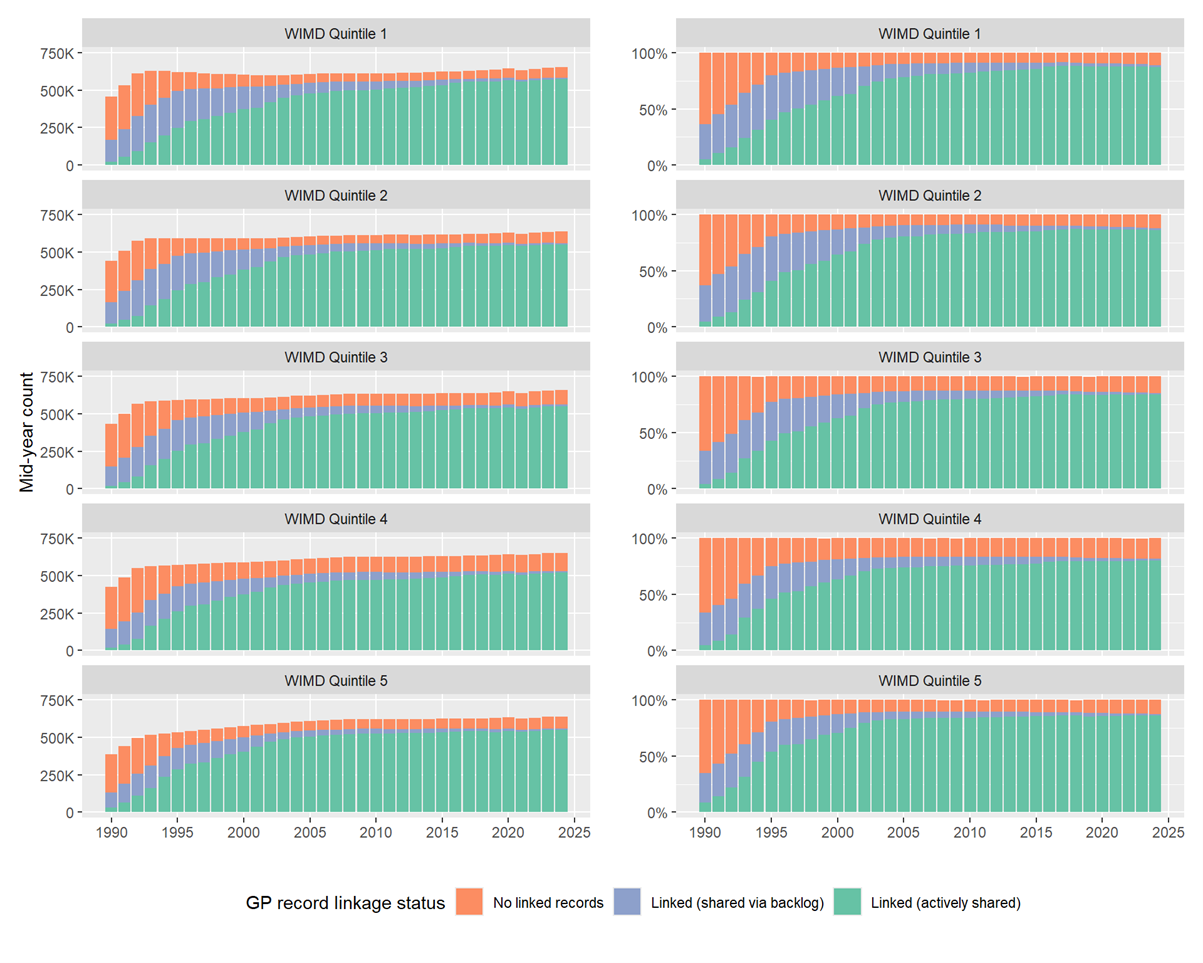

Supplement: S3 Fig — (TIF) [file pone.0338652.s007.tif]

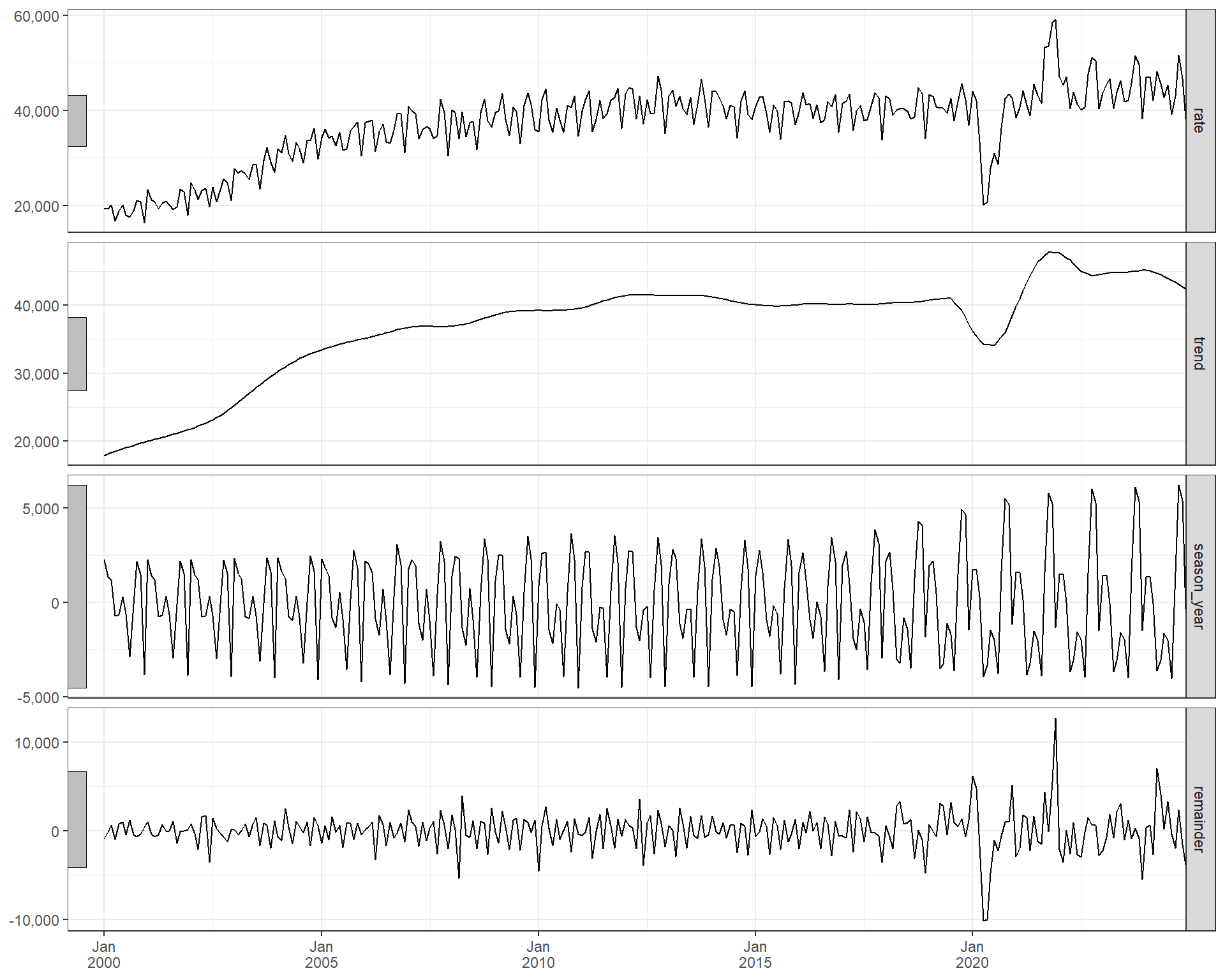

Supplement: S4 Fig — (TIF) [file pone.0338652.s008.tif]

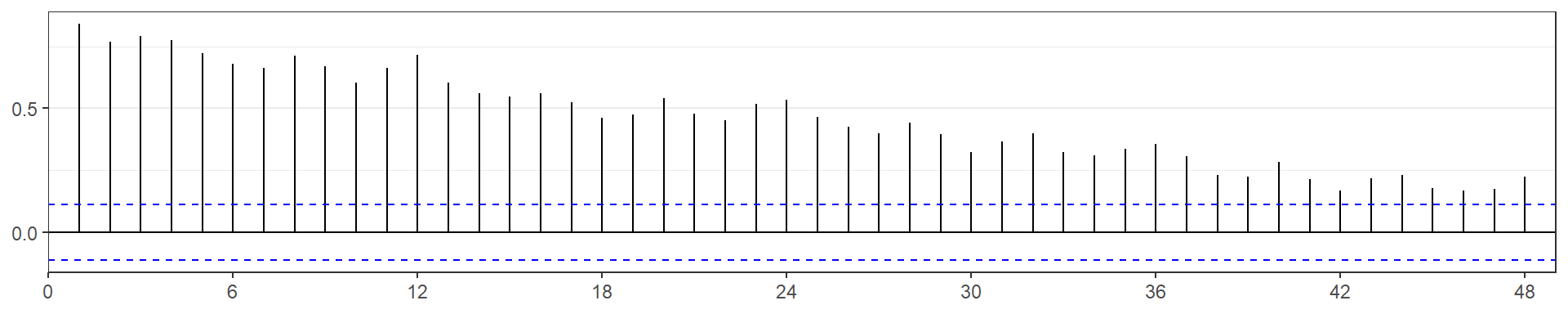

Supplement: S5 Fig — (TIF) [file pone.0338652.s009.tif]

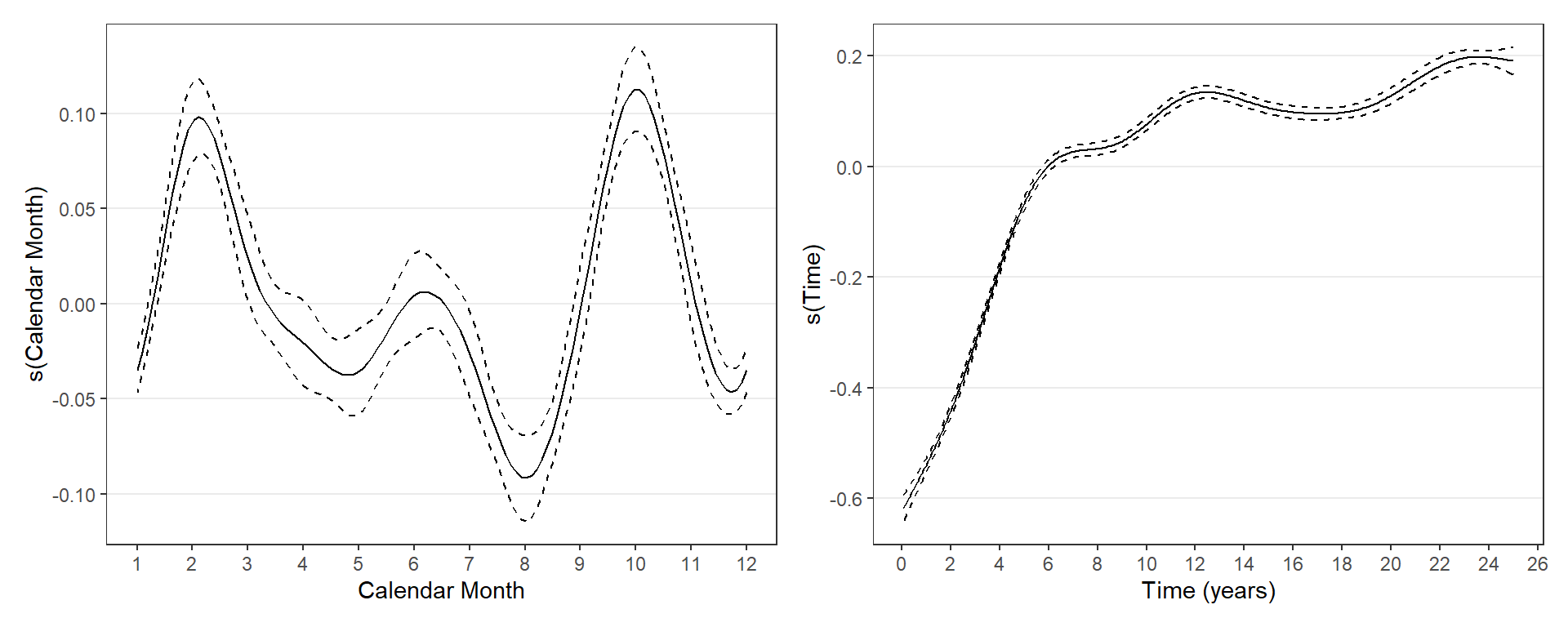

Supplement: S6 Fig — (TIF) [file pone.0338652.s010.tif]

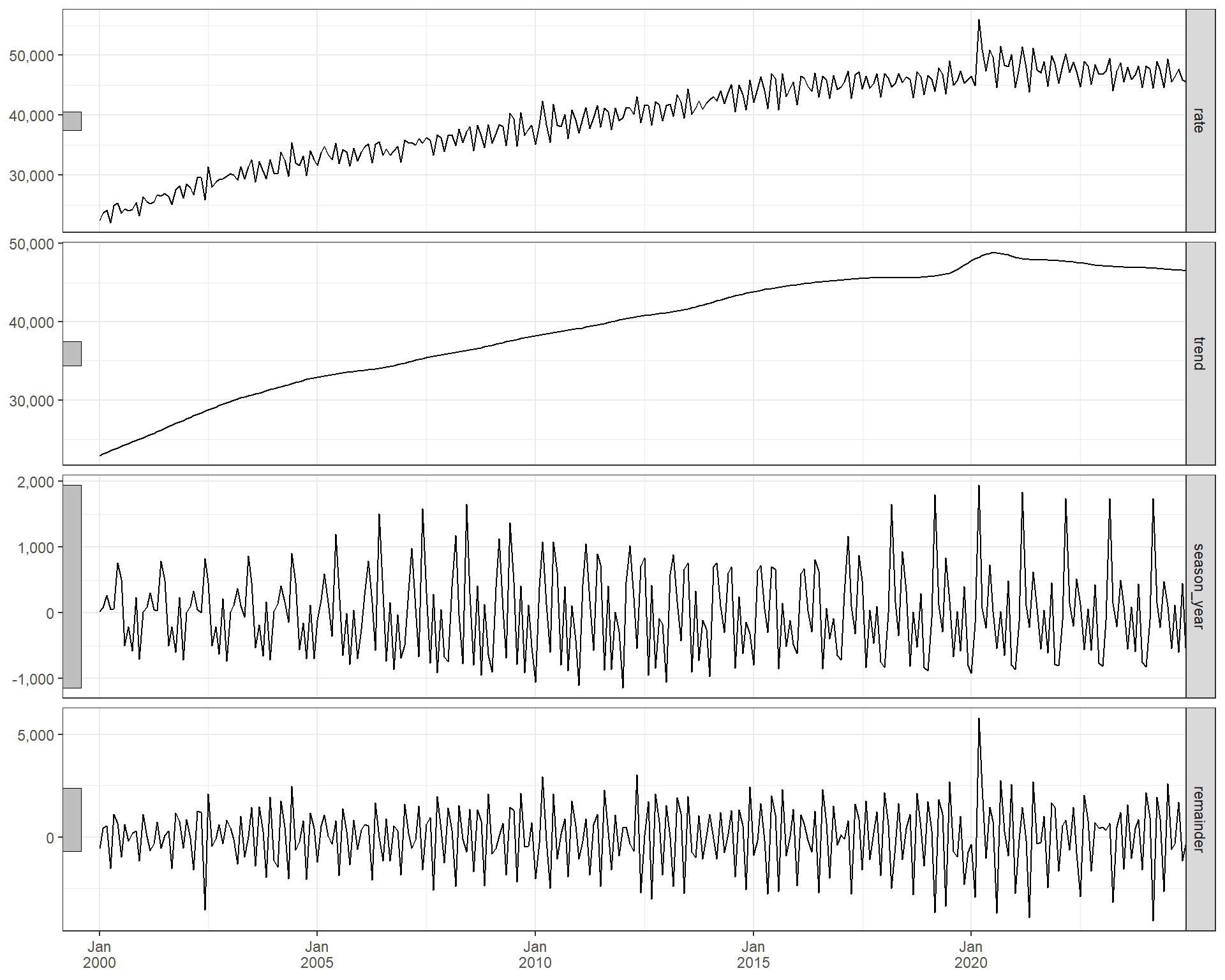

Supplement: S7 Fig — (TIF) [file pone.0338652.s011.tif]

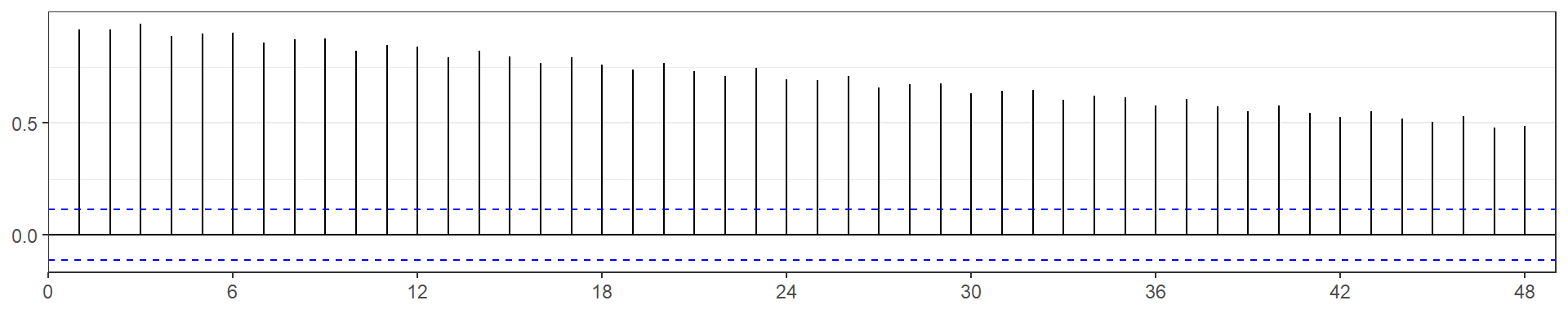

Supplement: S8Fig — (TIF) [file pone.0338652.s012.tif]

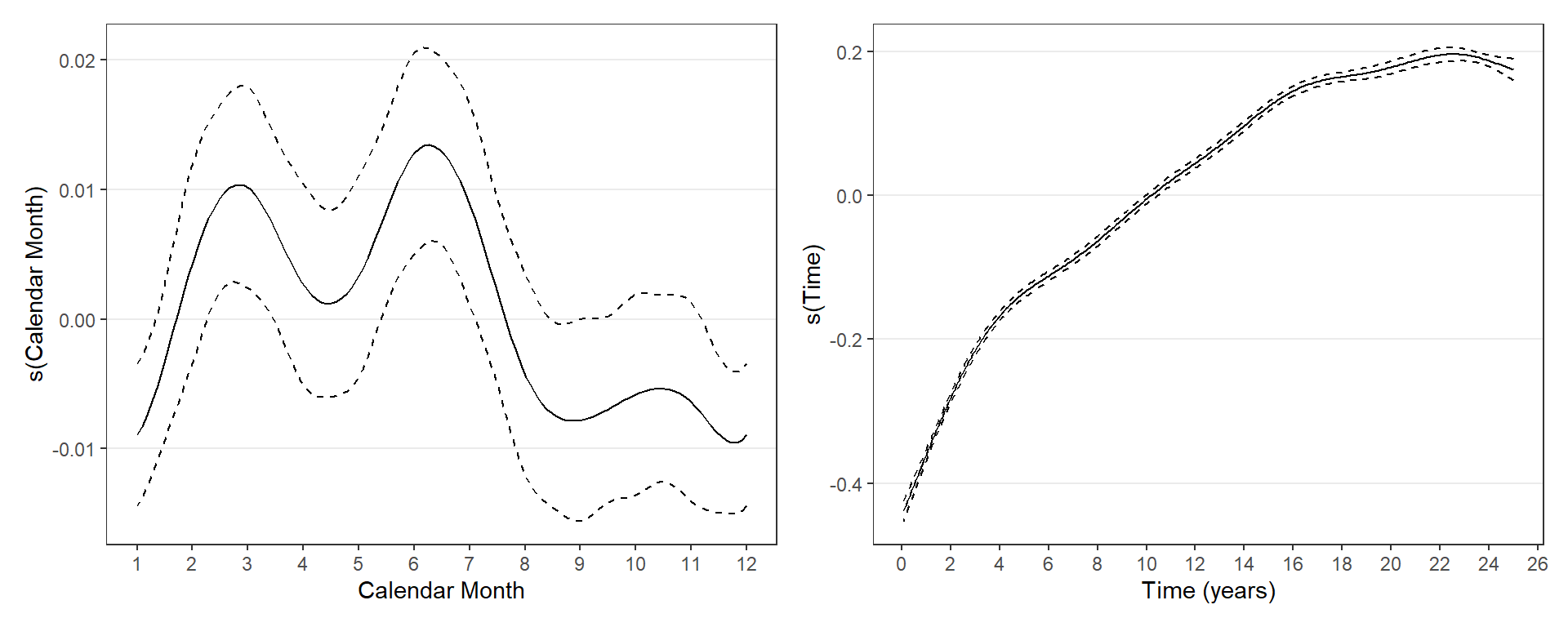

Supplement: S9 Fig — (TIF) [file pone.0338652.s013.tif]

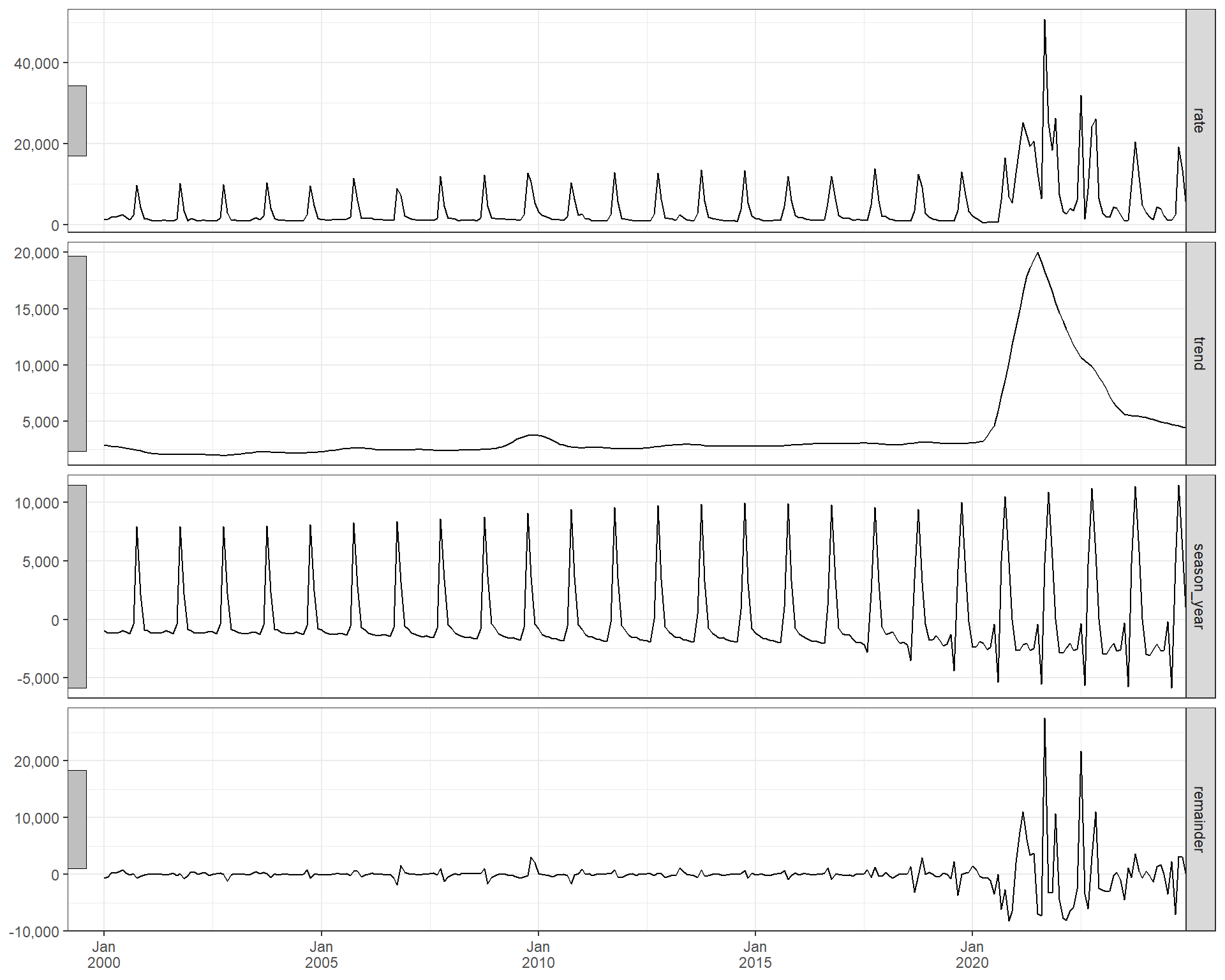

Supplement: S10 Fig — (TIF) [file pone.0338652.s014.tif]

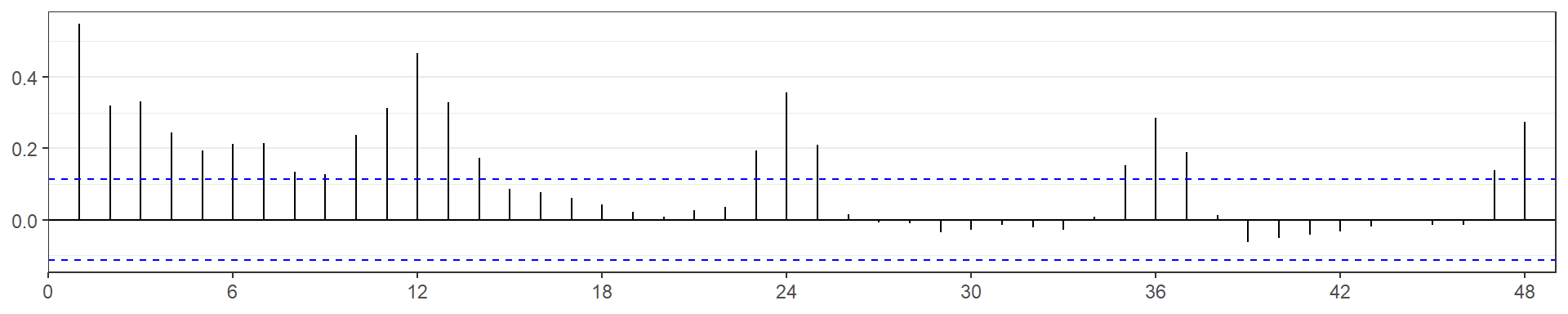

Supplement: S11 Fig — (TIF) [file pone.0338652.s015.tif]

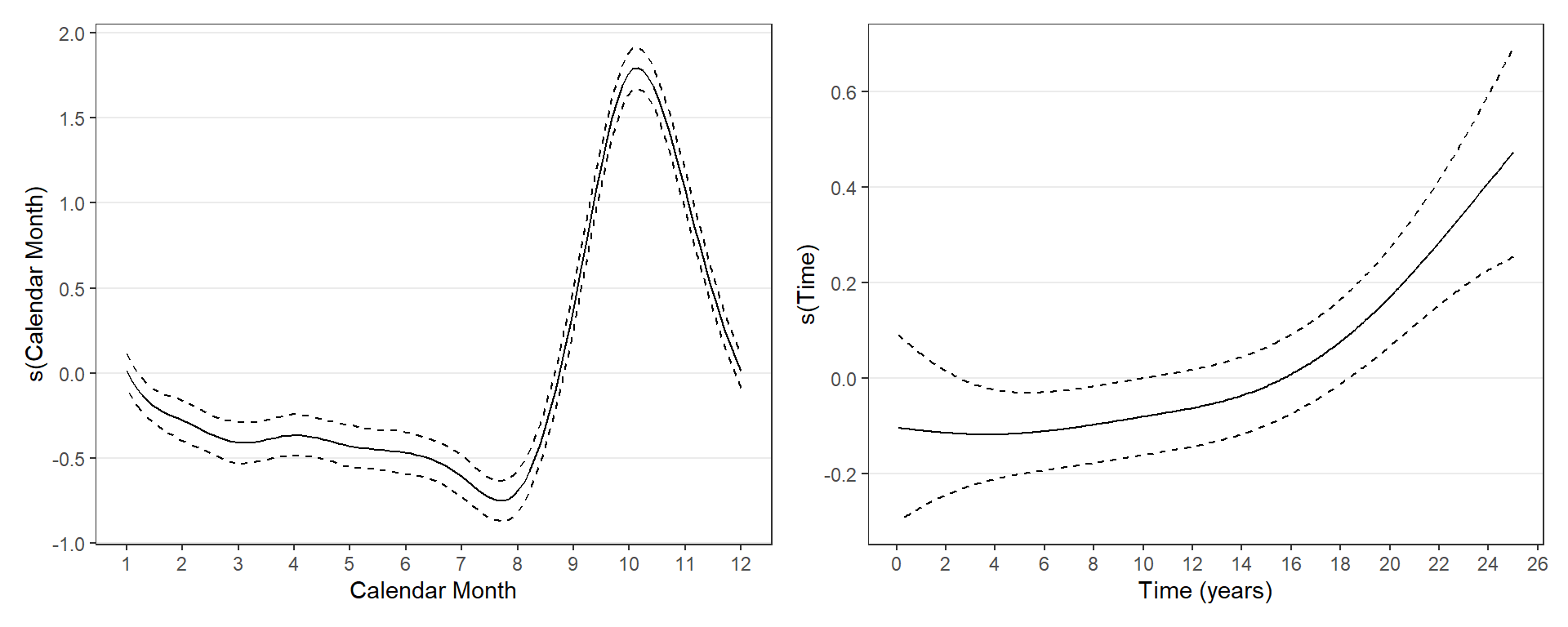

Supplement: S12 Fig — (TIF) [file pone.0338652.s016.tif]

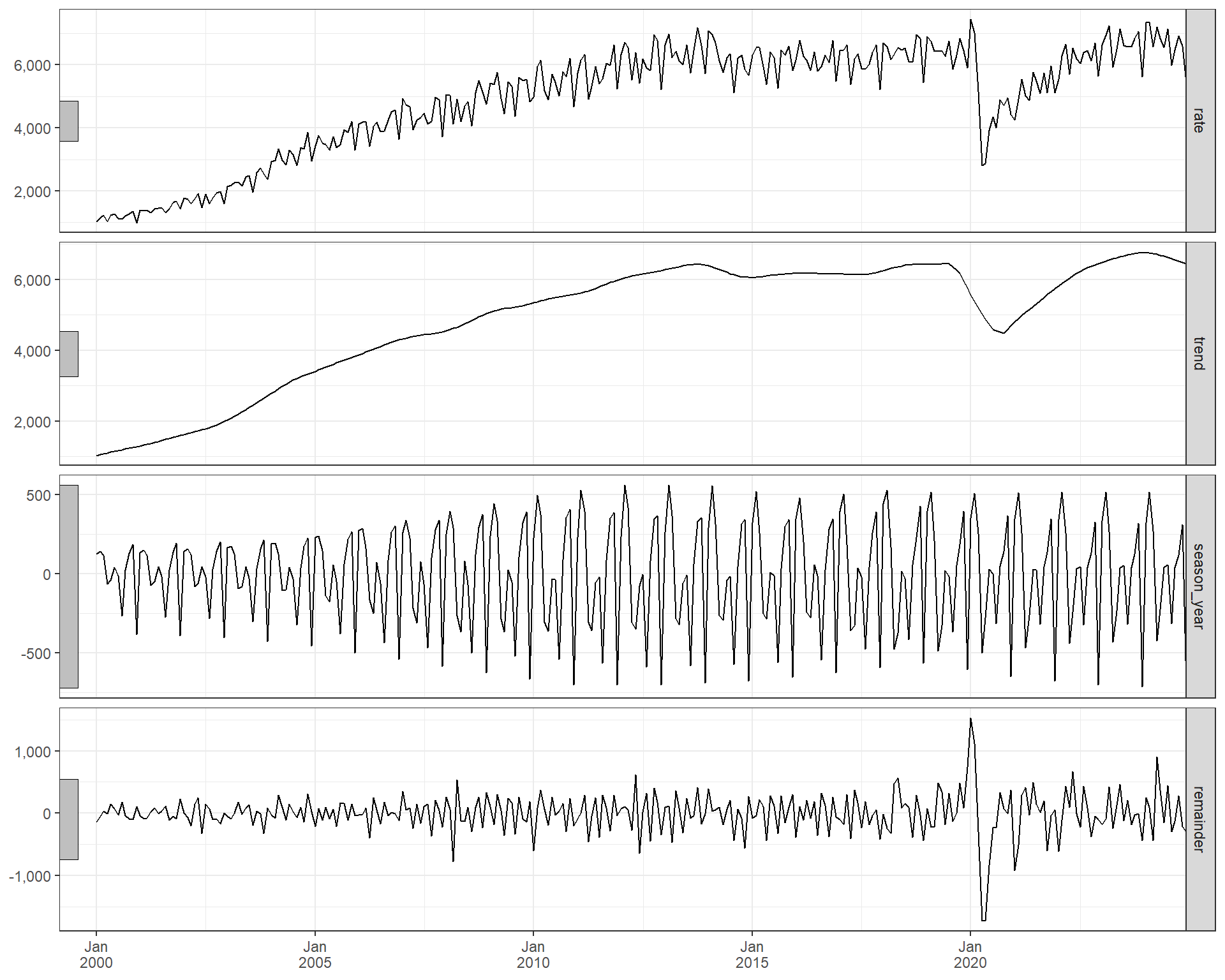

Supplement: S13 Fig — (TIF) [file pone.0338652.s017.tif]

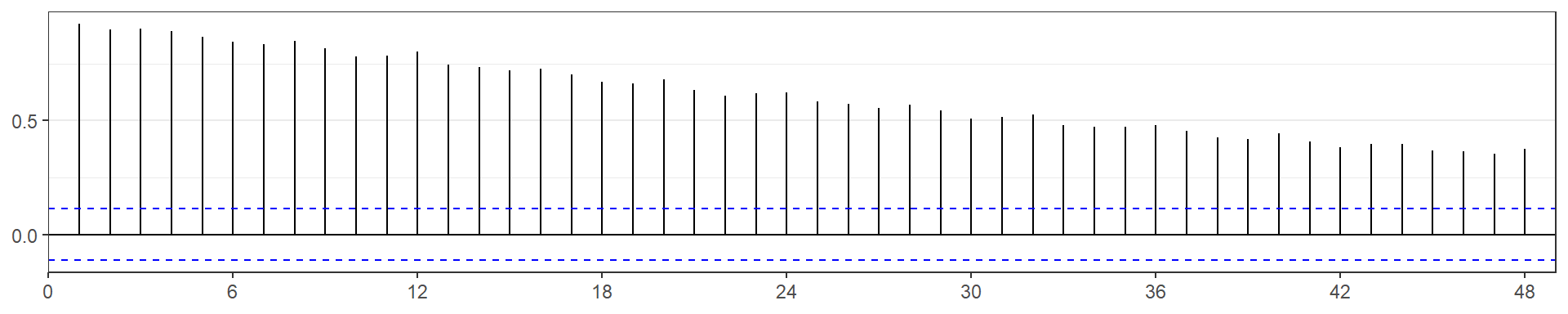

Supplement: S14 Fig — (TIF) [file pone.0338652.s018.tif]

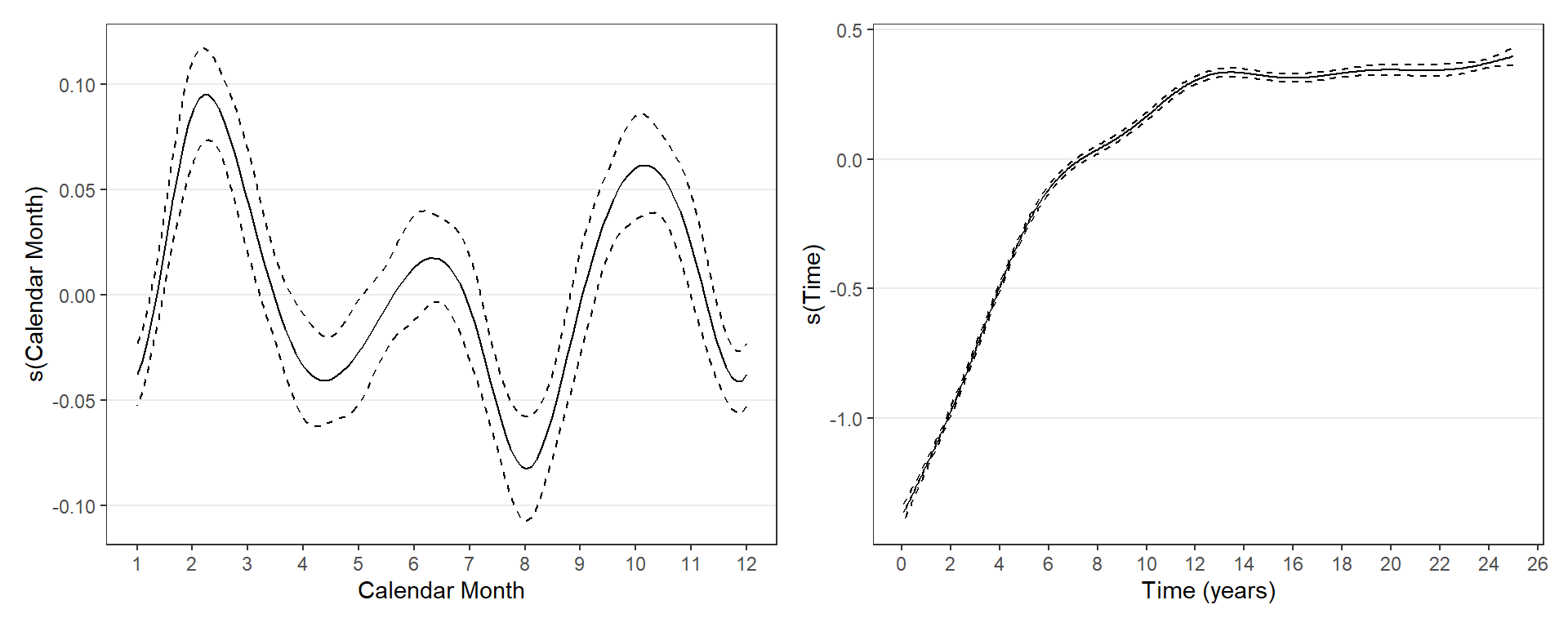

Supplement: S15 Fig — (TIF) [file pone.0338652.s019.tif]

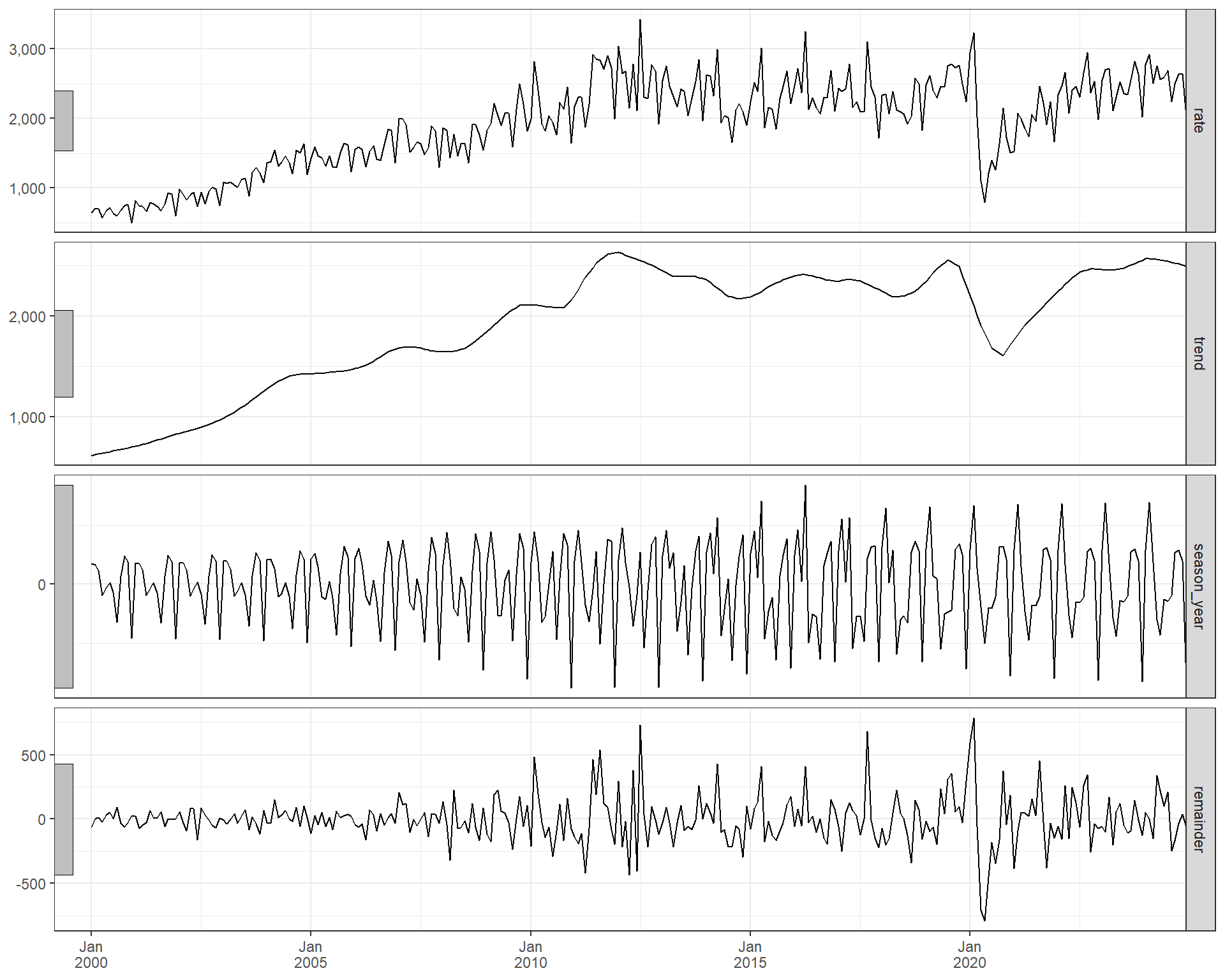

Supplement: S16 Fig — (TIF) [file pone.0338652.s020.tif]

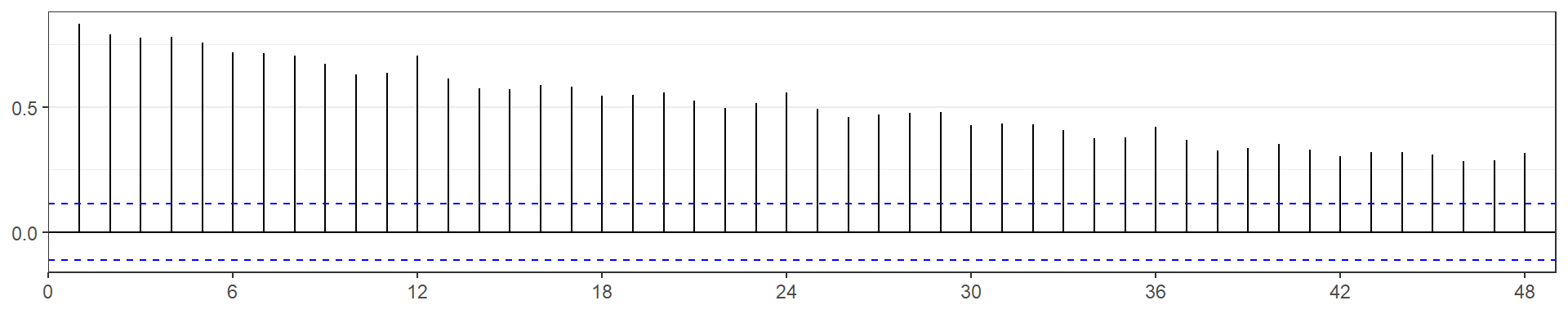

Supplement: S17 Fig — (TIF) [file pone.0338652.s021.tif]

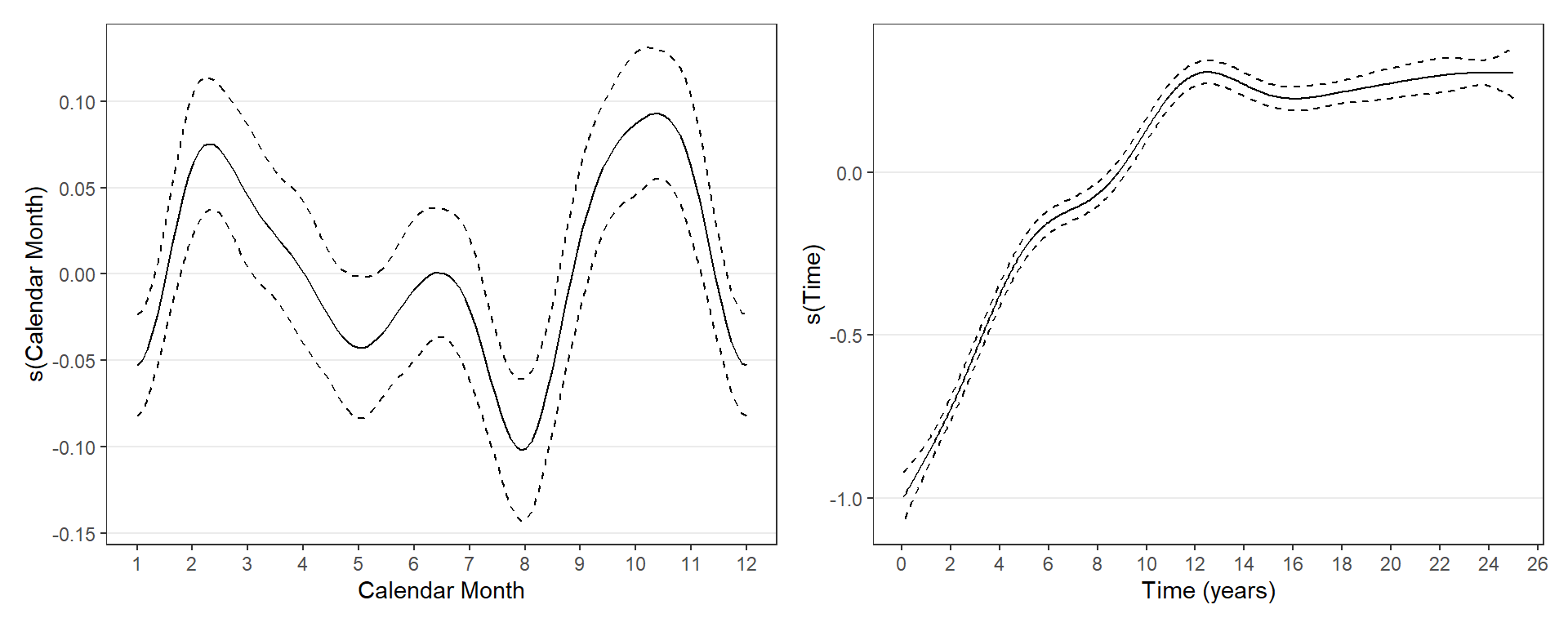

Supplement: S18 Fig — (TIF) [file pone.0338652.s022.tif]

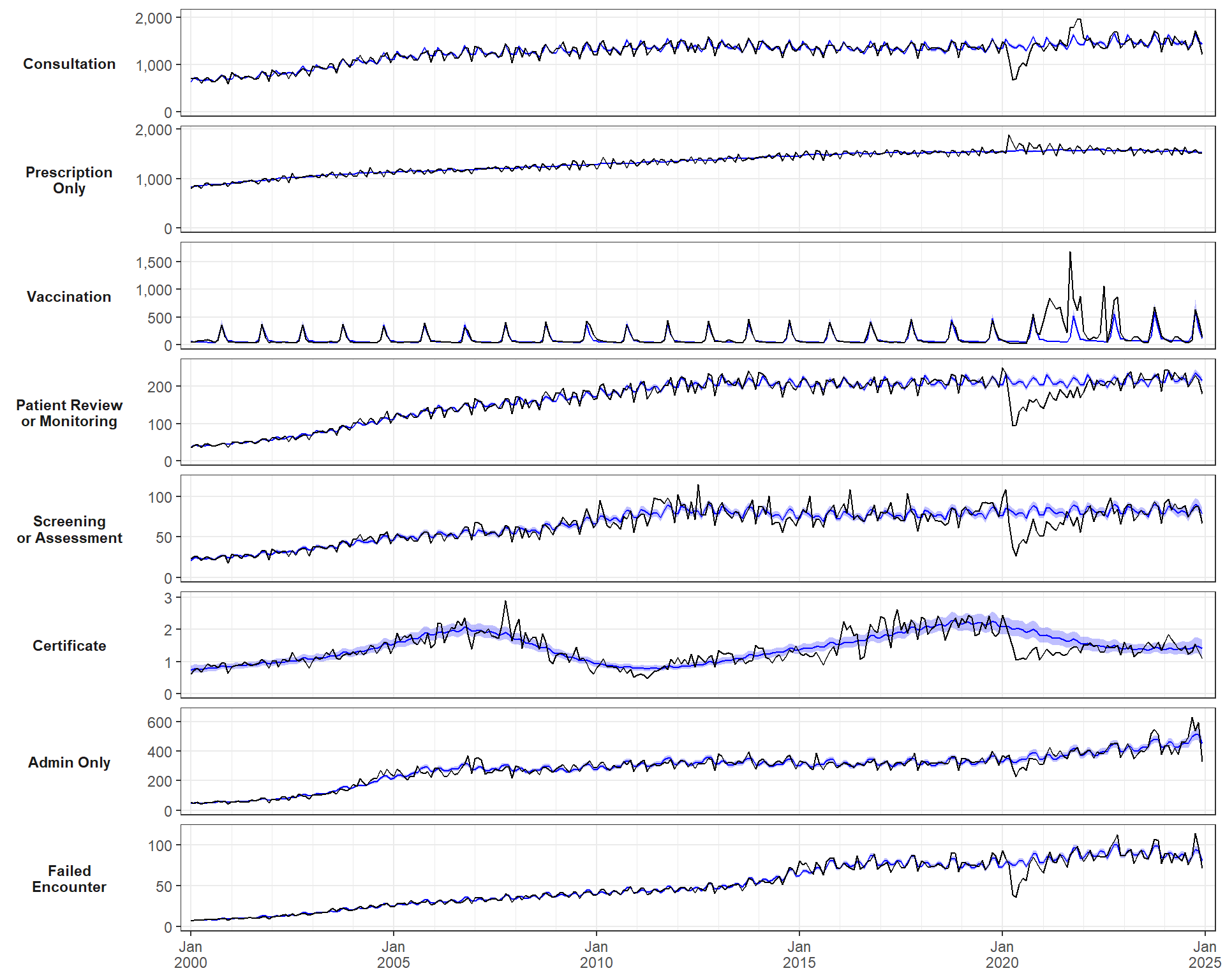

Supplement: S19 Fig — (TIF) [file pone.0338652.s023.tif]
