# Supplementary material for: Creating a Research-Ready Data Asset version of primary care data for Wales and investigating the impact of COVID-19 on utilisation of primary care services
Source: PLoS One. 2025 Dec 10;20(12):e0338652. doi: 10.1371/journal.pone.0338652 (PMC12694842; doi:10.1371/journal.pone.0338652)
Supplement: Attachment — Submitted filename: Response_to_Reviewers.docx [file pone.0338652.s025.docx]

**Title: Creating a Research-Ready Data Asset version of primary care data for Wales and investigating the impact of COVID-19 on utilisation of primary care services**

**Journal Requirements:**

When submitting your revision, we need you to address these additional requirements.

1. Please ensure that your manuscript meets PLOS ONE's style requirements, including those for file naming. The PLOS ONE style templates can be found at https://journals.plos.org/plosone/s/file?id=wjVg/PLOSOne_formatting_sample_main_body.pdf and https://journals.plos.org/plosone/s/file?id=ba62/PLOSOne_formatting_sample_title_authors_affiliations.pdf

**RESPONSE:** We confirm that the manuscript has been prepared in accordance with the PLOS ONE formatting and style requirements, and file naming conventions follow the journal’s guidelines.

2. We note that you have indicated that there are restrictions to data sharing for this study. For studies involving human research participant data or other sensitive data, we encourage authors to share de-identified or anonymized data. However, when data cannot be publicly shared for ethical reasons, we allow authors to make their data sets available upon request. For information on unacceptable data access restrictions, please see http://journals.plos.org/plosone/s/data-availability#loc-unacceptable-data-access-restrictions.

Before we proceed with your manuscript, please address the following prompts:

a) If there are ethical or legal restrictions on sharing a de-identified data set, please explain them in detail (e.g., data contain potentially identifying or sensitive patient information, data are owned by a third-party organization, etc.) and who has imposed them (e.g., a Research Ethics Committee or Institutional Review Board, etc.). Please also provide contact information for a data access committee, ethics committee, or other institutional body to which data requests may be sent.

b) If there are no restrictions, please upload the minimal anonymized data set necessary to replicate your study findings to a stable, public repository and provide us with the relevant URLs, DOIs, or accession numbers. Please see <http://www.bmj.com/content/340/bmj.c181.long> for guidelines on how to de-identify and prepare clinical data for publication. For a list of recommended repositories, please see <https://journals.plos.org/plosone/s/recommended-repositories>. You also have the option of uploading the data as Supporting Information files, but we would recommend depositing data directly to a data repository if possible.

Please update your Data Availability statement in the submission form accordingly.

**RESPONSE:** We confirm that our existing Data Availability statement already addresses these points. The data used in this study are held within the SAIL Databank, and access is subject to governance and approval by the independent Information Governance Review Panel (IGRP). As detailed in the statement, data cannot be publicly shared due to legal and ethical restrictions related to patient confidentiality, but researchers can request access through the SAIL application process (<https://saildatabank.com/data/apply-to-work-with-the-data/>). All relevant contact details, governance information, and guidance on data access are available on the SAIL Databank website (<https://saildatabank.com>).

3. Thank you for stating the following financial disclosure:
This work was supported by the ADR Wales programme of work. ADR Wales, part of the ADR UK investment, unites research expertise from Swansea University Medical School and WISERD (Wales Institute of Social and Economic Research and Data) at Cardiff University with analysts from Welsh Government. ADR UK is funded by the Economic and Social Research Council (ESRC), part of UK Research and Innovation. This research was supported by ESRC funding, including Administrative Data Research Wales (ES/W012227/1).

Please state what role the funders took in the study. If the funders had no role, please state: "The funders had no role in study design, data collection and analysis, decision to publish, or preparation of the manuscript."
If this statement is not correct you must amend it as needed.
Please include this amended Role of Funder statement in your cover letter; we will change the online submission form on your behalf.

**RESPONSE:** We confirm that the funders had no role in study design, data collection and analysis, decision to publish, or preparation of the manuscript. This statement has been added to the cover letter as requested.

4. We note you have included a table to which you do not refer in the text of your manuscript. Please ensure that you refer to Table 2 and 3 in your text; if accepted, production will need this reference to link the reader to the Table.

**RESPONSE:** We have reviewed the manuscript and ensured that all tables, including Tables 2 and 3, are appropriately referenced in the text.

5. If the reviewer comments include a recommendation to cite specific previously published works, please review and evaluate these publications to determine whether they are relevant and should be cited. There is no requirement to cite these works unless the editor has indicated otherwise.

**Additional Editor Comments:**

We have received feedback from two reviewers on your manuscript. They provide some useful suggestions on changes that should be made to improve the readibility and clarity of the manuscript. An important aspect that needs to be appropriately adressed is a specific description of how it is planned that this data based should be able to be accessed by other researchers; if this is the true ambition of the composition of the database. Please also make sure to correct errors in the references and the missing supplement information that reviewer 1 highlights.
**RESPONSE:** We confirm that all reviewer comments and editorial suggestions have been addressed, including corrections to references and the missing supplementary information.
Regarding data access, as detailed in the Data Availability statement, the data used in this study are held within the SAIL Databank and access is governed by the independent Information Governance Review Panel (IGRP). Due to legal and ethical restrictions related to patient confidentiality, data cannot be publicly shared, but researchers can request access through the SAIL application process (<https://saildatabank.com/data/apply-to-work-with-the-data/>). All relevant contact details, governance information, and guidance on data access are available on the SAIL Databank website (<https://saildatabank.com>).

We have also expanded the Discussion section to outline how future researchers can use or build upon the WLGP RRDA, including its potential for linkage with other datasets within SAIL to support broader health and care research.

**Reviewers' comments:**

**Reviewer #1:**

The paper describes a thorough and rigorous attempt to make a database that is usable for researching trends and healthcare utilisation in Primary Care. This is not something that is easy to do. I am not particularly familiar with GAM but as far as I can tell they appear to have been applied correctly.
Standard of English is good although there is a very extensive use of abbreviations, and these aren’t explained in Figure or table legends. The Results are confusing as they appear to contain some additional detail on the methods.

**RESPONSE:** Thank you for your positive and constructive feedback. We have added a dedicated abbreviations section and revised the manuscript to address all reviewer comments and improve clarity throughout.

I have made some specific comments below.
For readability consider a supplementary table containing abbreviations. Consider not using abbreviations when there are few occurrences e.g. Lower layer Super Output Areas (LSOA) only occurs once.

**RESPONSE:** We have added a section for abbreviations at the end of paper and have removed abbreviations which were used only once.

P8 Step 1 to P10 Step 4. I find the use of “record” confusing. I think that the authors are referring to database records as to complete patient records, but this should be clarified.

**RESPONSE:** We appreciate the reviewer’s comment and understand the potential for confusion. In this paper, the term record refers to a single event entry (i.e., a row) within the WLGP data rather than a complete patient record. As described earlier in the manuscript, “The WLGP data is structured in a long-format event-list format, which currently contains over 4.6 billion records.” To further clarify this meaning, we have now updated step 1 as follows:
“Records (i.e., individual event entries in the WLGP data) with missing ALF, event date, or practice ID were removed, as these are essential for identifying and linking patient data.”
We have also reviewed the section and replaced some instances of “record” with “event record” where appropriate to reinforce this context.

P15 Lines 330 to 332. “WLGP coverage was defined as the number of individuals with shared records”. It’s not clear to me how this defines coverage. Records shared with what?

**RESPONSE:** We agree more clarity can be added here, we have now updated the sentence to read:

“WLGP coverage was defined as the number of individuals living in Wales whose GP records were available within the SAIL Databank, either because their current GP practice at the time was actively sharing data with SAIL, or because historical records were later added through data shared by a future GP practice participating with SAIL.”

P18 Lines 385-388. Not sure why this text is here. It doesn’t seem like Results.

**RESPONSE:** We appreciate the reviewer’s observation. We agree that some of the content in the final paragraph of this section overlapped with the Methods description of the WLGP RRDA structure. We have therefore revised this paragraph to focus specifically on the observed benefits of the normalised structure—namely the improved query performance and analytical scalability—which are direct results of the RRDA design, and therefore appropriately belongs in the Results section. The revised paragraph reads as follows:
“Normalising the WLGP data as part of the RRDA development substantially improved query performance and analytical efficiency. The three-table relational format facilitated more scalable and iterative analysis compared to the raw format, particularly when working with longitudinal large time spans or full-population extracts.”

P18 Lines 390-396. Again I’m not sure that this is the right place for this paragraph. Much of it appears to be Method rather than result.

**RESPONSE:** Thanks for this helpful suggestion. We have moved the lines to the Method section, subsection ‘WLGP RRDA development’, under explanation of ‘Step 5 (Creation of a comprehensive look-up of primary care clinical codes)‘.

P26 Lines 540 to 544. It would be nice to see some more specific suggestions of how the database could be utilised, beyond the example of GP activity pre- and post-Covid.

**RESPONSE:** Thanks for the suggestion. We have expanded the last paragraph of the discussion to provide additional examples of how the WLGP RRDA can be utilised beyond the GP activity pre- and post-COVID analysis. Specifically, we now highlight that the RRDA can be linked, via the anonymised linkage field (ALF, the unique encrypted person identifier in SAIL), to a wide range of other individual-level datasets within the SAIL Databank (e.g., secondary care, demographic, maternity, and mortality data) to support research into patient pathways, outcomes, and healthcare system integration. The revised text also clarifies that the RRDA can be applied to a broad range of studies using SAIL data where primary care information is relevant. We have also added a paragraph to explain how the variation in data quality and completeness over time should be considered when designing longitudinal studies using this resource. These paragraphs now read as:
“Together, these findings highlight the value of a structured, reusable, and well-documented data asset for supporting timely and reliable research into primary care trends and outcomes in Wales. As with all individual-level data sources within the SAIL Databank, the WLGP RRDA includes the ALF, which enables secure linkage to other data sources such as secondary care, maternity and child health, mortality, census, and administrative data sources. This linkage capacity allows researchers to examine patient pathways and outcomes across the continuum of care. While our example focused on GP activity trends before and after the COVID-19 pandemic, the WLGP RRDA can be applied to a wide range of research questions where primary care data are relevant.

Building on these capabilities, the WLGP RRDA also enhances the usability of routine primary care data for research. Our evaluation provides insight into its strengths and limitations, particularly regarding data completeness and the potential impact of healthcare disruptions. When designing studies using the RRDA, researchers should carefully consider temporal coverage and population representativeness, as these factors may influence study findings. Although overall coverage is high, variation in which GP practices share data with SAIL could introduce selection bias. Two approaches can help address this. First, researchers can create inverse probability weights based on the likelihood of an individual having linked WLGP records. This would rebalance the study sample to better reflect the underlying population, as those with linked records but underrepresented characteristics receive greater weight in the analysis. Second, sensitivity analyses can be conducted by restricting studies to specific time periods or geographical regions with stable data coverage, then comparing these results with findings from the primary analytical approach to assess robustness.”

**Reviewer #2:**

This manuscript, 'Creating a Research-Ready Data Asset version of primary care data for Wales and investigating the impact of COVID-19 on utilisation of primary care services', presents a substantial piece of work. Some comments are as follows:

**INTRODUCTION**
There is some repetition around the description of online consultations. Lines 72-72 read “While these changes preserved access during lockdown, they also introduced questions about the long-term impact on patient care. And lines 76-77 read “While this preserved access during lockdowns, it also raised concerns about equity, diagnostic accuracy, and continuity of care.
**RESPONSE:** Thank you for this helpful comment. We have revised the text to remove repetition and to clarify the narrative structure.

Please clarify sentence at line 85: “At the same time, activity types in primary care also changed.” At the same time as what? The previous sentence mentions both before COVID-19 and during the pandemic.

**RESPONSE:** Thank you for the helpful comment. We have clarified this sentence to specify that the changes in activity types occurred during the COVID-19 pandemic, aligning it more clearly with the previous discussion of access mode changes. The revised sentence now reads: “The pandemic also led to substantial changes in the types of activities delivered in primary care.”

In line 138 the authors state that “the motivation behind this is twofold” and then say firstly… secondly…. and thirdly. The first two points describe motivations, while the third (“thirdly…”) seems to refer to an action taken rather than a motivating factor.

**RESPONSE:** Thanks for this helpful observation. We have revised the paragraph to clearly distinguish between the motivations for developing the RRDA and its applications.

**METHODS**
Lines 155-157 say: “The WLGP includes records for all patients registered with Welsh GPs, for the GPs who have agreed to share data with the SAIL Databank, as GPs are the data owners and must individually consent to contribute”. This link (<https://research.senedd.wales/research-articles/patient-record-sharing-clearing-up-the-confusion/>) however suggests individual patients may also be able to opt out: “Welsh patients can only opt-out by making a request to their GP”. Please clarify this in the text.

**RESPONSE:** Thanks for pointing this out. We have updated the text to clarify that, in addition to GP-level consent, individual patients can opt out of having their anonymised records included in SAIL by making a request to their GP. This aligns with SAIL’s guidance on opt-out procedures. The paragraph now starts with:
“We used anonymised individual-level linked data within the SAIL Databank to develop an efficient RRDA version for the WLGP data. The WLGP includes records for all patients registered with Welsh GPs, for the GPs who have agreed to share data with the SAIL Databank, as GPs are the data owners and must individually consent to contribute [23]. Furthermore, individual patients can opt out of having their anonymised records included in SAIL by making a request to their GP [24]. Currently, WLGP covers 86% of the Welsh population and 83% of GPs in Wales [25]. ...”

Line 163. Minor language/style suggestion: in scientific writing “data” is traditionally treated as a plural noun (i.e. “data are …”, “these data …”) especially in epidemiology / health data research. Suggest changing “data was” to “data were”. Please also check for use of “this data” in the rest of the manuscript (to change to “these data”).

**RESPONSE:** Thank you for this suggestion. We have reviewed the manuscript and updated the language accordingly, using plural forms where appropriate. In instances where “data” refers to a specific named dataset (e.g., “WLGP data”, or “primary care data”), we have retained the singular form.

Line 178. Suggest defining the abbreviation “SNOMED” at first use.

**RESPONSE:** Done.

Line 206: The sentence describing the WDSD practice ID reads a bit awkwardly “(called WDSD practice ID in the paper)”. Consider rephrasing, although, looking through the manuscript, I can only find use of “practice ID” and not “WDSD practice ID”, so perhaps this detail is not needed?

**RESPONSE:** We have removed the phrase “(called WDSD practice ID in the paper)” as it was not used elsewhere in the manuscript.

Line 223: What is meant by “For any codes used in WLGP that did not fall into one of these categories (i.e., unverified or invalid codes), we created a new category and included them in the look-up”? Could you clarify what this new category was called?

**RESPONSE:** We have replaced the statement with the following to clarify (which was moved here from the Results section): “In addition to the recognised code types, we included two supplementary categories in the clinical code look-up: ‘Blank or invalid codes’, referring codes that were blank or failed basic format validation; and ‘Unknown codes’, which appeared structurally valid but lacked known descriptions or classification, often originating from legacy or undocumented local codes used by software providers, or codes set up by practices themselves. The inclusion of these supplementary categories allowed us to classify all clinical events in the RRDA and enhanced the interpretability of this data.”

Lines 228-229. The authors standardised the format of additional Vision codes to the 5-character level, either by removing extra characters or adding a "." to the right if the code was shorter than five characters. Could they clarify if the new standardized codes were unique i.e. is there a risk that two originally distinct codes could become identical after truncation? The whole of step 5 is slightly complicated. Consider providing some examples of how codes were standardised, truncated/padded, or prioritised, with descriptions, in the supplementary material.

**RESPONSE:** We confirm that cases where two codes became identical after truncation or padding were handled systematically through the prioritisation process already described in the manuscript, where the first code type encountered was retained based on the predefined hierarchy (official Read V2 → SNOMED → local Vision → local EMIS → additional Read/Vision → EMIS from the UK Biobank). We have clarified the text in *Step 5* to make it explicit that duplicate codes could arise not only from truncation or padding but also from overlapping entries across different data sources (e.g. EMIS codes present in both the DHCW and UK Biobank lists). The revised paragraph now explains that all such duplicates were resolved using a predefined prioritisation hierarchy based on code type:

“Some challenges arose during the creation of the look-up table. For instance, we encountered cases where the same SNOMED-CT and Vision local codes had different descriptions. To address this, we standardised the format of Vision codes to the 5-character level by either removing extra characters or padding with a “.” to the right when shorter than five characters. A similar approach was applied to EMIS and Vision codes. We also found overlap between Read V2 and EMIS codes, which we resolved by treating overlapping codes as Read codes and removing them from the EMIS list. In general, to manage duplicate codes across different code types —whether arising from truncation or padding, or from overlapping entries across sources (e.g. EMIS codes appearing both in DHCW and UK Biobank lists)— we prioritised the codes based on their type, retaining the first code type encountered in the following order: official Read V2 codes, official SNOMED codes, DHCW local Vision codes, DHCW local EMIS codes, additional Read or Vision codes, and additional EMIS codes from the UK Biobank. See S1 File in the Supporting Infromation some examples.”

As suggested, we have added a short illustrative table in the supporting information providing examples of how clinical codes were standardised, truncated, or prioritised during look-up creation. These examples are illustrative and do not include any real or sensitive data.

Figure 1. Consider briefly clarifying in the figure legend or a note that the “foreign key” refers back to the primary key in another table (to help readers unfamiliar with database terminology).

**RESPONSE:** Thanks for your suggestion. We have updated the figure legend as follows:
“**Fig 1: Structure of normalised WLGP RRDA tables.** A primary key uniquely identifies each row in a table, while a foreign key is a column in one table that links to the primary key in another table. ”

Line 259: The paragraph in the Methods section beginning with “Understanding how patients interact with primary care services…” reads more like a rationale or background statement than a methodological description. As this is the Methods section, it would be clearer to focus on the procedural aspects of what was done.

**RESPONSE:** Thanks for this helpful observation. We have removed the first sentence of the section ‘Multi-layer approach for identifying types of patient-practice interactions’ as this was already explained in the Introduction.

Line 288: Please clarifying why SNOMED codes were excluded.

**RESPONSE:** SNOMED codes were excluded because, while there are more than 1 m SNOMED codes, they account for only a small proportion of records in the WLGP RRDA (approximately 0.1%). Moreover, the SNOMED coding system has a substantially different hierarchical structure compared with Read codes, meaning that developing equivalent classification rules would require considerable effort with limited added value for this dataset. The revised text in the Methods section (Multi-layer approach for identifying types of patient-practice interactions) now explains this rationale:
“To implement this system, we leveraged hierarchies and categories of both official and local clinical codes (where available), along with free-text search, to ensure accurate classification while maintaining flexibility for evolving clinical documentation practices. For this part of the work, we included only official Read V2 codes, local EMIS and Vision codes, additional Read or Vision codes, and supplementary EMIS codes from the UK Biobank. SNOMED codes were excluded from this classification step as they account for only 0.1% of WLGP RRDA records and use a different hierarchical structure from Read codes, with over one million codes, making their inclusion disproportionately complex for limited benefit. Overall, this classification approach enhances the interpretability of primary care records and provides a robust framework for analysing patient-provider interactions at scale.”

Lines 315-316: Where additional primary care person-day event categories were defined, are these mutually exclusive, or can a single event belong to multiple categories?

**RESPONSE:** The additional person-day event categories are not necessarily mutually exclusive; a single event may meet the criteria for more than one category (e.g., an event could involve both “Patient review or monitoring” and “Certificate issuance”). We have added a clarifying sentence after the list of categories to make this explicit.

Line 323: It would be helpful to clarify what is meant by “failed encounters.” For example, could this refer to patients who did not attend scheduled appointments, cancelled appointments, or other types of incomplete interactions?

**RESPONSE:** We have expanded the definition of “failed encounters” to clarify that it includes instances where a patient did not attend or was not brought to a scheduled appointment, where an appointment was cancelled by either the patient or the healthcare provider, where the patient could not be contacted, or where an invitation or consultation was declined by the patient. The revised definition has been added to the manuscript for clarity and now reads as follows:

“Failed encounters: Events exclusively containing a record of a failed primary care encounter, defined as instances where a patient did not attend or was not brought to a scheduled appointment, where an appointment was cancelled by either the patient or the healthcare provider, where the patient could not be contacted, or where an invitation or consultation was declined by the patient.”

Line 334: It would be helpful to explicitly state the unit of analysis in the Generalised Additive Mixed Models. I note at the end of the paragraph it says a fuller exploratory analysis is provided in Section 1 of the Supplementary Material, but I could only find supporting tables and figures, not “Section 1”.

**RESPONSE:** We have edited the text to now explicitly state the unit of analysis being one calendar month. And regarding the supplementary exploratory analyses, we now refer to the supplementary Tables and Figures directly:
“Supplementary Tables S1-S3 and Figures S1-S3 contain an expanded set of descriptive counts...”

And:

“Supplementary Figure S4-S19 contains results from the initial an exploratory analysis of the GP activity trends...”
Line 336: Can you define AR(1) (e.g., “autoregressive of order 1”) when it is first introduced.

**RESPONSE:** We’ve edited the relevant sentence to now read:
“...the general trend over the years, and an autoregressive covariance structure of order 1, AR(1), between year-on-year observations...”

with the rest of the sentence explains the purpose of the AR(1) structure.

Line 347: Please also specify the unit of analysis for this analysis.

**RESPONSE:** We’ve edited the relevant sentence to now read:

“The unit of analysis was one calendar month, with the outcome being the average daily number of events that month per 100,000 people for a given activity.”

Lines 355-357: COVID-19 disruption periods are mentioned, but it might help to explain why vaccination exclusions are slightly different (2020–2021 vs 2021–2022).

**RESPONSE:** Thanks for asking for explanation, as we have now noticed we go the year ranges the wrong way round. We have now corrected this as well as added an explanation to the text:

“We omitted observations from 2020 and 2021 from model fitting due to COVID-19 pandemic restrictions and disruptions. For vaccinations, we excluded 2021 and 2022 instead, as this period coincided with the rollout of the UK-wide COVID-19 vaccination programme. This caused a huge increase in vaccination activity over the two-year period, as population subgroups became eligible for the initial two-dose schedule, as well as booster doses.”

**RESULTS**
Line 395: Please correct link (Error! Reference source not found).

**RESPONSE:** Thanks, we have fixed the link.

Line 399. When referring to/describing the Figure in the text, please say whether you are referring to panel a) or b). The caption for Figure 4 could be clarified/expanded to specify what the percentages are calculated from. For example, I think the bars represents the % of unique clinical codes not in the official Read V2 code set / the % of total records using such codes.

**RESPONSE:** We have clarified all references in the text to specify whether we are referring to panel (A) or (B). We also revised the Figure 4 legend to specify how the percentages were calculated—namely, the proportion of distinct codes or records in each year that were coded using non-Read V2 code types (e.g., EMIS, Vision, SNOMED) in the WLGP RRDA.

Line 419-420: (Error! Reference source not found.)
Line 430: (Error! Reference source not found.)

**RESPONSE:** Thanks, we have fixed both links.

Line 446: The figure title includes “(WIMD 2019)” but since the plot also includes other variables (age, sex, and health board), is (WIMD 2019) needed in the title or could it be removed? Could a note be included under the figure to say what has been adjusted for in the adjusted ORs?

**RESPONSE:** We have removed ‘(WIMD 2019)’ from the figure title and updated the caption so it now reads:
“Fig 6: Unadjusted and adjusted odds ratios of having linked GP records. Adjusted estimates were obtained from multivariable models controlling for all other covariates under study.”

Line 462-463: The text says, “For GP events in which only a prescription was recorded, we saw a steady increase in the daily rates from 21,543 to 44,568 (+207%) between January 2000 and December 2024”. Is the 207% correct? Should it be 107% increase?

**RESPONSE:** Thanks, we’ve rephrased this as a “+107%”, and referred to any decreases as negatives e.g. 0.8 times the expected value will be written as “–20%”.

Line 467-468: Similar to the comment above, the text says, “Daily rate of vaccinations was always highest in October, and up until the COVID-19 pandemic had been slightly increasing over time, 9,680 in October 2000 to 13,759 in October 2019 (+142%)”. Is 142% correct? Should it be 42% increase? Or it could be phrased 1.42 times higher?

**RESPONSE:** We’ve rephrased this as “+42%”, along with all other similar occurrences.

**DISCUSSION**
Can the authors make recommendations for future researchers who may want to use this resource? For example, could the RRDA be linked with secondary care datasets to enable more comprehensive analyses of patient pathways and outcomes. Additionally, given the variation in data quality/completeness over time, how this should be considered when designing longitudinal studies using this resource?

**RESPONSE:** We have expanded the last paragraphs of the discussion to now highlight that the RRDA can be applied to a broad range of studies using SAIL data where primary care information is relevant, that the RRDA can be linked to a wide range of other individual-level data sets, we also provide additional examples of how the WLGP RRDA can be utilised, as well as clarify that care should be taken when designing a study utilising this data source:

“Together, these findings highlight the value of a structured, reusable, and well-documented data asset for supporting timely and reliable research into primary care trends and outcomes in Wales. As with all individual-level data sources within the SAIL Databank, the WLGP RRDA includes the ALF, which enables secure linkage to other data sources such as secondary care, maternity and child health, mortality, census, and administrative data sources. This linkage capacity allows researchers to examine patient pathways and outcomes across the continuum of care. While our example focused on GP activity trends before and after the COVID-19 pandemic, the WLGP RRDA can be applied to a wide range of research questions where primary care data are relevant.

Building on these capabilities, the WLGP RRDA also enhances the usability of routine primary care data for research. Our evaluation provides insight into its strengths and limitations, particularly regarding data completeness and the potential impact of healthcare disruptions. When designing studies using the RRDA, researchers should carefully consider temporal coverage and population representativeness, as these factors may influence study findings. Although overall coverage is high, variation in which GP practices share data with SAIL could introduce selection bias. Two approaches can help address this. First, researchers can create inverse probability weights based on the likelihood of an individual having linked WLGP records. This would rebalance the study sample to better reflect the underlying population, as those with linked records but underrepresented characteristics receive greater weight in the analysis. Second, sensitivity analyses can be conducted by restricting studies to specific time periods or geographical regions with stable data coverage, then comparing these results with findings from the primary analytical approach to assess robustness.”
